# Supplementary material for: Altered Gut Microbiota Composition Is Associated with Difficulty in Explicit Emotion Regulation in Young Children
Source: Microorganisms. 2023 Sep 6;11(9):2245. doi: 10.3390/microorganisms11092245 (PMC10535925; doi:10.3390/microorganisms11092245)
Supplement: Supplementary file 1 [file microorganisms-11-02245-s001.zip › microorganisms-2509899-supplementary.pdf]

SUPPLEMENTARY MATERIAL

Altered gut microbiota composition is associated with difficulty in explicit emotion regulation in young children

Section S1: Analysis of demographic data

For comparison of the categorical variables of sex, family income, and older siblings, we performed Fisher's exact test with the R basic function Fisher test. We used the Mann–Whitney *U* test to compare the age and mother's years of schooling, as normality was unmet. The Brunner–Munzel test was performed to compare BMI as normality and homoscedasticity were not met.

Supplementary Tables

Table S1. *Definitions from Behavior Rating Inventory of Executive Function—Preschool Version (BRIEF-P)*

Items	Description
**Broader index**	
Inhibitory Self-Control (ISC)	Measure to represent the child's ability to adapt emotion and behavior with appropriate inhibitory control. The index comprises the subscales "Inhibition" and "Emotion Control." ISC is considered an index related to EER as it contains "Emotion Control" in its subscale. ISC difficulties are associated with externalizing (e.g., aggression and antisociality) and internalizing (e.g., anxiety and depression) problems in daily life [75].
Flexibility (FL)	Represents the ability to shift flexibly between behavior and emotions. The index comprises the subscales "Shifting" and "Emotion Control." FL is considered to be a part within EER.
Emergent Metacognition (EM)	Quantification of the ability to solve problems through planning while holding ideas and efforts to complete the task in working memory. This is the only one of the three broader scales that does not have emotional or behavioral regulation in subscale items, and this index is composed of the subscales "Working Memory" and "Planning." Working Memory and Planning subscales contribute to more cognitive aspects and are consistent with the concept of CC [32]. Thus, EM can be considered an index of CC. CC difficulties are associated with lower task performance in laboratory experiments related to CC (e.g., 6-boxes task, [76]).
**Subscale index**	
Inhibition	Controls impulses and behavior; appropriately stops and modulates own behavior at the proper time or in the proper context
Emotion Control	Modulates emotional responses appropriately to situational demand or context
Working Memory	Holds information in mind for the purpose of completing a task or making the appropriate response; stays with or sticks to an activity
Shifting	Moves freely from one situation, activity, or aspect of a problem to another as the situation demands; makes transitions; solves problems flexibly

Planning	Anticipates future events or consequences; uses goals or instructions to guide behavior in context; develops or implements appropriate steps ahead of time to conduct an associated task or action

Note. From "BRIEF-P interceptive report," by Isquith et al. [77].

Table S2. *Subscale and broader scale scores on BRIEF-P*

	Full sample	ISC		FL		EM	
		Risk	Non-Risk	Risk	Non-Risk	Risk	Non-Risk
**Broader index**[a]							
Inhibitory Self-control (α = .90)	1.35 (0.29)	1.95 (0.19)	1.28 (0.22)	1.92 (0.28)	1.31 (0.25)	1.80 (0.31)	1.31 (0.26)
Flexibility (α = .85)	1.28 (0.25)	1.68 (0.28)	1.23 (0.20)	1.89 (0.17)	1.23 (0.19)	1.55 (0.32)	1.25 (0.23)
Emergent Metacognition (α = .75)	1.33 (0.27)	1.70 (0.32)	1.29 (0.23)	1.66 (0.35)	1.30 (0.25)	1.96 (0.17)	1.27 (0.20)
**Subscale index**[a]							
Inhibition (α = .90)	1.40 (0.35)	2.07 (0.31)	1.32 (0.27)	1.87 (0.44)	1.36 (0.32)	1.98 (0.42)	1.35 (0.30)
Emotion Control (α = .80)	1.30 (0.30)	1.83 (0.31)	1.24 (0.24)	1.98 (0.26)	1.25 (0.24)	1.63 (0.37)	1.27 (0.28)
Working Memory (α = .87)	1.31 (0.29)	1.69 (0.34)	1.26 (0.25)	1.65 (0.34)	1.28 (0.27)	1.93 (0.28)	1.25 (0.22)
Shifting (α = .77)	1.25 (0.27)	1.54 (0.34)	1.22 (0.24)	1.81 (0.31)	1.21 (0.21)	1.48 (0.37)	1.23 (0.25)
Planning (α = .75)	1.35 (0.28)	1.72 (0.34)	1.31 (0.24)	1.67 (0.41)	1.33 (0.26)	2.00 (0.18)	1.29 (0.21)

Note. Cronbach's α was obtained by the following formula: α = k/k-1(1-Σσi2/σx2).
α: coefficient alpha; k: number of items; σx2: Total variance of the scale; σi2: variance of the item.
[a]Mean (SD).

Table S3. *Composition of the number of participants in the EF-risk, EF non-risk, and EF-control groups*

**ISC-risk group**				**ISC non-risk group**			
ISC-risk only	ISC-risk ∩ FL-risk	ISC-risk ∩ EM-risk	ISC-risk ∩ FL-risk ∩ EM-risk	FL-risk only	EM-risk only	FL-risk ∩ EM-risk	No applicable risks **(i.e., ISC-control group)**
10	7	5	4	4	8	3	216
**FL-risk group**				**FL non-risk group**			
FL-risk only	FL-risk ∩ EM-risk	FL-risk ∩ ISC-risk	FL-risk ∩ EM-risk ∩ ISC-risk	EM-risk only	ISC-risk only	EM-risk ∩ ISC-risk	No applicable risks **(i.e., FL-control group)**
4	3	7	4	8	10	5	216
**EM-risk group**				**EM non-risk group**			
EM-risk only	EM-risk ISC-risk	EM-risk ∩ FL-risk	EM-risk ∩ ISC-risk ∩ FL-risk	ISC-risk only	FL-risk only	ISC-risk ∩ FL- risk	No applicable risks **(i.e., EM-control group)**
8	5	3	4	10	4	7	216

Note. Table S3 describes the type of risk and number of children in each EF-risk, and EF non-risk, EF-control group. For example, the ISC risk group included children at ISC risk only (n=10), ISC and FL risk (n=7), ISC and EM risk (n=5), and all at risk (ISC, FL, EM) (n=4). The ISC non-risk group included children at FL risk only (n=4), EM risk only (n=8), FL and EM risk (n=3), and not risks applicable (n=216).
Abbreviations: ISC, Inhibitory Self-Control; FL, Flexibility; EM, Emergent Metacognition; ∩, Intersection.

Table S4. *Definitions from the Bristol Stool Scale*

**Score**	**Description**
1	Separate hard lumps, like nuts (hard to pass)
2	Sausage-shaped but lumpy
3	Like a sausage, but with cracks on the surface
4	Like a sausage or snake, smooth and soft
5	Soft blobs with clear-cut edges (passes easily)
6	Fluffy pieces with ragged edges, a mushy stool
7	Watery, no solid pieces, entirely liquid

Note. From [43]. The Bristol Stool Scale categorizes defecation patterns as follows: scores 1 and 2 are indicative of constipation, scores 3–5 fall within the range of normal defecation, while scores 6 and 7 signify diarrhea [43]. Based on previous research [43, 44], we categorized scores 1, 2, 6, and 7 as abnormal.

Table S5. *List of genus-level bacteria with a prevalence greater than 25%*

Phylum	Class	Order	Family	Genus
Actinobacteria	*Actinobacteria*	*Actinomycetales*	*Actinomycetaceae*	*Actinomyces*
Actinobacteria	*Actinobacteria*	*Bifidobacteriales*	*Bifidobacteriaceae*	*Bifidobacterium*
Actinobacteria	*Coriobacteriia*	*Coriobacteriales*	*Coriobacteriaceae*	*Collinsella*
Actinobacteria	*Coriobacteriia*	*Coriobacteriales*	*Coriobacteriaceae*	*Eggerthella*
Actinobacteria	*Coriobacteriia*	*Coriobacteriales*	*Coriobacteriaceae*	*Gordonibacter*
Bacteroidetes	*Bacteroidia*	*Bacteroidales*	*Bacteroidaceae*	*Bacteroides*
Bacteroidetes	*Bacteroidia*	*Bacteroidales*	*Marinifilaceae*	*Odoribacter*
Bacteroidetes	*Bacteroidia*	*Bacteroidales*	*Prevotellaceae*	*Prevotella*
Bacteroidetes	*Bacteroidia*	*Bacteroidales*	*Rikenellaceae*	*Alistipes*
Bacteroidetes	*Bacteroidia*	*Bacteroidales*	*Tannerellaceae*	*Parabacteroides*
Firmicutes	*Bacilli*	*Bacillales*	*Bacillaceae*	*Bacillus*
Firmicutes	*Bacilli*	*Erysipelotrichales*	*Erysipelatoclostridiaceae*	*Coprobacillus*
Firmicutes	*Bacilli*	*Erysipelotrichales*	*Erysipelatoclostridiaceae*	*Erysipelatoclostridium*
Firmicutes	*Bacilli*	*Erysipelotrichales*	*Erysipelotrichaceae*	*Faecalitalea*
Firmicutes	*Bacilli*	*Erysipelotrichales*	*Erysipelotrichaceae*	*Holdemania*
Firmicutes	*Bacilli*	*Erysipelotrichales*	*Erysipelotrichaceae*	*Turicibacter*
Firmicutes	*Bacilli*	*Lactobacillales*	*Carnobacteriaceae*	*Granulicatella*
Firmicutes	*Bacilli*	*Lactobacillales*	*Enterococcaceae*	*Enterococcus*
Firmicutes	*Bacilli*	*Lactobacillales*	*Lactobacillaceae*	*Lactobacillus*
Firmicutes	*Bacilli*	*Lactobacillales*	*Streptococcaceae*	*Streptococcus*
Firmicutes	*Clostridia*	*Clostridiales*	*Clostridiaceae*	*Clostridium*
Firmicutes	*Clostridia*	*Clostridiales*	*Clostridiaceae*	*Intestinibacter*
Firmicutes	*Clostridia*	*Clostridiales*	*Clostridiaceae*	*Romboutsia*
Firmicutes	*Clostridia*	*Clostridiales*	*Lachnospiraceae*	*Agathobacter*
Firmicutes	*Clostridia*	*Clostridiales*	*Lachnospiraceae*	*Anaerostipes*
Firmicutes	*Clostridia*	*Clostridiales*	*Lachnospiraceae*	*Blautia*
Firmicutes	*Clostridia*	*Clostridiales*	*Lachnospiraceae*	*Dorea*
Firmicutes	*Clostridia*	*Clostridiales*	*Lachnospiraceae*	*Eisenbergiella*
Firmicutes	*Clostridia*	*Clostridiales*	*Lachnospiraceae*	*Fusicatenibacter*
Firmicutes	*Clostridia*	*Clostridiales*	*Lachnospiraceae*	*Hungatella*
Firmicutes	*Clostridia*	*Clostridiales*	*Lachnospiraceae*	*Lachnoclostridium*
Firmicutes	*Clostridia*	*Clostridiales*	*Lachnospiraceae*	*Lachnospira*
Firmicutes	*Clostridia*	*Clostridiales*	*Lachnospiraceae*	*Roseburia*
Firmicutes	*Clostridia*	*Clostridiales*	*Lachnospiraceae*	*Sellimonas*

Firmicutes	*Clostridia*	*Clostridiales*	*Lachnospiraceae*	*Tuzzerella*
Firmicutes	*Clostridia*	*Clostridiales*	*Lachnospiraceae*	*Tyzzerella*
Firmicutes	*Clostridia*	*Clostridiales*	*Lachnospiraceae*	*UCG-001*
Firmicutes	*Clostridia*	*Clostridiales*	*Lachnospiraceae*	*UCG-004*
Firmicutes	*Clostridia*	*Clostridiales*	*Oscillospiraceae*	*Colidextribacter*
Firmicutes	*Clostridia*	*Clostridiales*	*Oscillospiraceae*	*Flavonifractor*
Firmicutes	*Clostridia*	*Clostridiales*	*Oscillospiraceae*	*Oscillibacter*
Firmicutes	*Clostridia*	*Clostridiales*	*Oscillospiraceae*	*Oscillospira*
Firmicutes	*Clostridia*	*Clostridiales*	*Ruminococcaceae*	*Anaerotruncus*
Firmicutes	*Clostridia*	*Clostridiales*	*Ruminococcaceae*	*Butyricicoccus*
Firmicutes	*Clostridia*	*Clostridiales*	*Ruminococcaceae*	*Candidatus Soleaferrea*
Firmicutes	*Clostridia*	*Clostridiales*	*Ruminococcaceae*	*Faecalibacterium*
Firmicutes	*Clostridia*	*Clostridiales*	*Ruminococcaceae*	*Negativibacillus*
Firmicutes	*Clostridia*	*Clostridiales*	*Ruminococcaceae*	*Ruminococcus*
Firmicutes	*Clostridia*	*Clostridiales*	*Ruminococcaceae*	*Subdoligranulum*
Firmicutes	*Clostridia*	*Clostridiales*	*Ruminococcaceae*	*UBA1819*
Firmicutes	*Clostridia*	*Monoglobales*	*Monoglobaceae*	*Monoglobus*
Firmicutes	*Negativicutes*	*Veillonellales*	*Veillonellaceae*	*Dialister*
Firmicutes	*Negativicutes*	*Veillonellales*	*Veillonellaceae*	*Veillonella*
Proteobacteria	*Deltaproteobacteria*	*Desulfovibrionales*	*Desulfovibrionaceae*	*Bilophila*
Proteobacteria	*Gammaproteobacteria*	*Burkholderiales*	*Sutterellaceae*	*Parasutterella*
Proteobacteria	*Gammaproteobacteria*	*Burkholderiales*	*Sutterellaceae*	*Sutterella*
Proteobacteria	*Gammaproteobacteria*	*Enterobacteriales*	*Enterobacteriaceae*	*Escherichia-Shigella*
Proteobacteria	*Gammaproteobacteria*	*Pasteurellales*	*Pasteurellaceae*	*Haemophilus*
Verrucomicrobia	*Verrucomicrobiae*	*Verrucomicrobiales*	*Verrucomicrobiaceae*	*Akkermansia*

Table S6. *Demographic details of all ISC-risk and ISC non-risk groups*

Items	All participants	ISC-risk group	ISC non-risk group	*p*-value	*q*-value
Sample (N)	257	26	231		
Antibiotic treatment within the last 3 months (N)	32 (13%)	3	29		
Age (months) [a]	46.53 (6.33)	46.04 (6.32)	46.59 (6.34)	0.72	0.81
Sex (boy/girl)	152/105	19/7	133/98	0.15	0.51
BMI [a]	15.76 (1.29)	16.43 (1.89)	15.69 (1.20)	0.08	0.51
Family income [b]	less than 3 million yen: 21 (8%)<br>3-5 million yen: 71 (30%)<br>5-7 million yen: 80 (34%)<br>more than 7 million yen: 65 (27%)<br>Not answered: 20	less than 3 million yen: 1 (4%)<br>3-5 million yen: 8 (33%)<br>5-7 million yen: 11 (46%)<br>more than 7 million yen: 4 (17%)<br>Not answered: 2	less than 3 million yen: 20 (9%)<br>3-5 million yen: 63 (30%)<br>5-7 million yen: 69 (32%)<br>more than 7 million yen: 61 (29%)<br>Not answered: 18	0.46	0.80
Mother's years of schooling [a]	14.77 (1.88)	14.50 (2.08)	14.80 (1.86)	0.29	0.68
Siblings [b]	Yes: 210 (83%)<br>No: 45 (17%)<br>Not answered: 2	Yes: 21 (81%)<br>No: 5 (19%)<br>Not answered: 0	Yes: 189 (83%)<br>No: 40 (17%)<br>Not answered: 2	0.79	0.81
Birth mode [b]	Vaginal delivery: 201 (78%)<br>C-section: 56 (22%)	Vaginal delivery: 20 (77%)<br>C-section: 6 (23%)	Vaginal delivery: 181 (78%)<br>C-section: 50 (22%)	0.81	0.81

[a]Mean (SD). [b] N (%)

Table S7. *Comparison of diversity of the microbiota between ISC-risk (N = 26) and ISC non-risk (N = 231) groups (ANCOVA, PERMANOVA)*

Alpha diversity	**All analyzed data**	**ISC-risk group**	**ISC non-risk group**	***F***	***p*-value**	***q*-value**	***η2G***
Shannon [a]	5.83 (0.49)	5.85 (0.61)	5.83 (0.48)	0.095	0.76	0.89	0.0004
Chao1 [a]	169.75 (44.07)	172.63 (50.82)	169.42 (43.35)	0.096	0.76	0.89	0.0004
Faith's PD [a]	22.42 (4.88)	22.74 (5.08)	22.39 (4.87)	0.104	0.75	0.89	0.0004
Observed species [a]	169.54 (44.04)	172.54 (50.88)	169.21 (43.32)	0.105	0.75	0.89	0.0004
Beta diversity				***F***	***p*-value**	***q*-value**	***R2***
ISC risk vs. ISC non-risk				0.663	0.78	0.89	0.0026

Note. Antibiotic treatment within the last 3 months and sex were used as covariates.
[a]Mean (SD)

Table S8. *Comparison of relative abundance of the microbiota between ISC-risk (N = 26) and ISC non-risk (N = 231) groups (ANCOM-BC)*

Genus	beta	SE	W	*p*-value	*q*-value	95% CI	
						low	high
Actinomyces	0.982	0.254	3.872	**1.08E-04*** **	**0.006** **	0.485	1.479
Bifidobacterium	0.039	0.145	0.268	0.788	0.890	-0.245	0.322
Collinsella	0.398	0.597	0.667	0.505	0.890	-0.772	1.568
Eggerthella	-0.042	0.208	-0.201	0.841	0.890	-0.449	0.366
Gordonibacter	0.112	0.200	0.562	0.574	0.890	-0.279	0.504
Bacteroides	0.069	0.107	0.644	0.520	0.890	-0.140	0.278
Odoribacter	0.549	0.451	1.215	0.224	0.812	-0.336	1.433
Prevotella	0.099	0.455	0.217	0.828	0.890	-0.793	0.990
Alistipes	-0.029	0.549	-0.053	0.958	0.928	-1.106	1.048
Parabacteroides	0.047	0.588	0.080	0.936	0.923	-1.106	1.200
Bacillus	0.586	0.409	1.434	0.151	0.782	-0.215	1.387
Coprobacillus	0.117	0.389	0.300	0.764	0.890	-0.645	0.878
Erysipelatoclostridium	0.261	0.368	0.709	0.479	0.890	-0.461	0.983
Faecalitalea	-0.190	0.380	-0.500	0.617	0.890	-0.936	0.555
Holdemania	-0.239	0.151	-1.584	0.113	0.680	-0.534	0.057
Turicibacter	-0.006	0.392	-0.016	0.988	0.928	-0.774	0.761
Granulicatella	0.375	0.336	1.116	0.265	0.838	-0.284	1.034
Enterococcus	0.446	0.359	1.240	0.215	0.812	-0.259	1.150
Lactobacillus	-0.352	0.394	-0.892	0.373	0.890	-1.124	0.421
Streptococcus	0.118	0.291	0.406	0.685	0.890	-0.453	0.689
Clostridium	0.496	0.421	1.179	0.238	0.812	-0.329	1.321
Intestinibacter	0.006	0.360	0.017	0.986	0.928	-0.700	0.712
Romboutsia	0.237	0.362	0.654	0.513	0.890	-0.473	0.947
Agathobacter	0.120	0.573	0.209	0.835	0.890	-1.004	1.244
Anaerostipes	-0.158	0.299	-0.527	0.598	0.890	-0.743	0.428
Blautia	-0.685	0.415	-1.652	0.099	0.680	-1.498	0.128
Dorea	0.293	0.425	0.690	0.490	0.890	-0.540	1.127
Eisenbergiella	0.069	0.376	0.184	0.854	0.890	-0.667	0.806
Fusicatenibacter	0.525	0.449	1.168	0.243	0.812	-0.356	1.406
Hungatella	0.231	0.330	0.699	0.484	0.890	-0.416	0.878
Lachnoclostridium	0.266	0.209	1.274	0.203	0.812	-0.143	0.675
Lachnospira	-0.842	0.414	-2.033	**0.042***	0.661	-1.653	-0.030
Roseburia	-0.766	0.429	-1.789	0.074	0.680	-1.606	0.073
Sellimonas	-0.037	0.346	-0.107	0.915	0.917	-0.714	0.640
Tuzzerella	-0.487	0.296	-1.648	0.099	0.680	-1.067	0.092
Tyzzerella	0.149	0.483	0.309	0.757	0.890	-0.798	1.096

Lachnospiraceae_UCG-001	0.409	0.297	1.376	0.169	0.782	-0.174	0.992
Lachnospiraceae_UCG-004	-0.196	0.399	-0.490	0.624	0.890	-0.978	0.587
Colidextribacter	0.140	0.356	0.395	0.693	0.890	-0.557	0.838
Flavonifractor	-0.041	0.261	-0.157	0.875	0.892	-0.553	0.471
Oscillibacter	0.182	0.341	0.534	0.594	0.890	-0.487	0.851
Oscillospira	-0.166	0.262	-0.634	0.526	0.890	-0.681	0.348
Anaerotruncus	-0.031	0.171	-0.179	0.858	0.890	-0.366	0.305
Butyricicoccus	-0.569	0.344	-1.653	0.098	0.680	-1.243	0.106
Candidatus Soleaferrea	0.197	0.215	0.913	0.361	0.890	-0.225	0.619
Faecalibacterium	0.134	0.298	0.448	0.654	0.890	-0.451	0.718
Negativibacillus	-0.128	0.247	-0.519	0.604	0.890	-0.612	0.356
Ruminococcus	-0.444	0.569	-0.781	0.435	0.890	-1.559	0.670
Subdoligranulum	-0.385	0.445	-0.864	0.388	0.890	-1.257	0.488
UBA1819	-0.513	0.316	-1.623	0.105	0.680	-1.132	0.107
Monoglobus	-0.894	0.444	-2.015	**0.044***	0.661	-1.764	-0.024
Dialister	-0.093	0.403	-0.232	0.817	0.890	-0.883	0.696
Veillonella	0.133	0.590	0.225	0.822	0.890	-1.023	1.289
Bilophila	0.102	0.386	0.265	0.791	0.890	-0.655	0.859
Parasutterella	-0.662	0.479	-1.382	0.167	0.782	-1.601	0.277
Sutterella	1.545	0.517	2.989	**3.E-03***	**0.084**†	0.532	2.558
Escherichia-Shigella	-0.199	0.548	-0.363	0.717	0.890	-1.274	0.876
Haemophilus	-0.109	0.478	-0.228	0.819	0.890	-1.046	0.827
Akkermansia	0.143	0.277	0.517	0.605	0.890	-0.400	0.687

Note. Antibiotic treatment within the last 3 months and sex were used as covariates.
*** $p < 0.001$; ** p or $q < 0.01$; * $p < 0.05$; $^{\dagger}q < 0.10$; beta, coefficient obtained from the ANCOM-BC log-linear model; se, standard error of beta; W, test statistic (beta/se)

Table S9. *Demographic details between the FL-risk and FL non-risk groups*

**Items**	**All participants**	**FL-risk group**	**FL-control group**	***p*-value**	***q*-value**
Sample (N)	257	18	239		
Antibiotic treatment within the last 3 months (N)	32	3	29 (13%)		
Age (months) [a]	46.53 (6.33)	44.56 (6.46)	46.68 (6.31)	0.17	0.24
Sex (boy/girl)	152/105	15/3	137/102	**0.04***	0.13
BMI [a]	15.76 (1.29)	16.35 (1.18)	15.71 (1.29)	0.06	0.13
Family income [b]	less than 3 million yen: 21 (8%)<br>3-5 million yen: 71 (30%)<br>5-7 million yen: 80 (34%)<br>more than 7 million yen: 65 (27%)<br>Not answered: 20	less than 3 million yen: 0 (0%)<br>3-5 million yen: 9 (56%)<br>5-7 million yen: 5 (31%)<br>more than 7 million yen: 2 (13%)<br>Not answered: 2	less than 3 million yen: 21 (10%)<br>3-5 million yen: 62 (28%)<br>5-7 million yen: 75 (34%)<br>more than 7 million yen: 63 (29%)<br>Not answered: 18	0.10	0.18
Mother's years of schooling [a]	14.77 (1.88)	14.72 (2.40)	14.77 (1.87)	0.46	0.54
Siblings [b]	Yes: 210 (82%)<br>No: 45 (18%)<br>Not answered: 2	Yes: 10 (56%)<br>No: 8 (44%)<br>Not answered: 0	Yes: 200 (84%)<br>No: 37 (16%)<br>Not answered: 2	**0.01***	**0.04***
Birth mode [b]	Vaginal delivery: 201 (78%)<br>C-section: 56 (22%)	Vaginal delivery: 15 (83%)<br>C-section: 3 (17%)	Vaginal delivery: 186 (78%)<br>C-section: 53 (22%)	0.77	0.77

* p or $q < 0.05$
[a]Mean (SD). [b] N (%)

Table S10. *Comparison of diversity of the microbiota between FL-risk (N = 18) and FL non-risk (N = 239) groups (ANCOVA, PERMANOVA)*

Alpha diversity	**All analyzed data**	**FL-risk group**	**FL non-risk group**	***F***	***p*-value**	***q*-value**	***η2G***
Shannon [a]	5.83 (0.49)	5.76 (0.56)	5.83 (0.49)	0.246	0.620	0.94	0.001
Chao1 [a]	169.75 (44.07)	160.91 (43.48)	170.41 (43.13)	0.803	0.371	0.79	0.003
Faith's PD [a]	22.42 (4.88)	21.76 (4.34)	22.47 (4.92)	0.348	0.556	0.92	0.001
Observed species [a]	169.54 (44.04)	160.50 (43.52)	170.23 (44.09)	0.836	0.361	0.79	0.003
Beta diversity				***F***	***p*-value**	***q*-value**	***R2***
FL risk vs. FL non-risk				1.521	0.104	0.67	0.006

Note. Antibiotic treatment within the last 3 months and sex were used as covariates.
[a]Mean (SD)

Table S11. *Comparison of relative abundance of the microbiota between FL-risk (N = 18) and FL non-risk (N = 239) groups (ANCOM-BC)*

**Genus**	**beta**	**se**	**W**	***p*-value**	***q*-value**	**95% CI**	
						**low**	**high**
Actinomyces	0.440	0.303	1.451	0.147	0.722	-0.154	1.034
Bifidobacterium	-0.149	0.164	-0.910	0.363	0.791	-0.470	0.172
Collinsella	-0.616	0.665	-0.926	0.355	0.791	-1.919	0.688
Eggerthella	0.079	0.192	0.410	0.682	0.970	-0.298	0.456
Gordonibacter	-0.455	0.202	-2.246	**0.025***	0.452	-0.851	-0.058
Bacteroides	0.149	0.124	1.203	0.229	0.791	-0.094	0.392
Odoribacter	0.460	0.512	0.898	0.369	0.791	-0.544	1.465
Prevotella	-0.442	0.486	-0.909	0.363	0.791	-1.394	0.510
Alistipes	-0.464	0.683	-0.679	0.497	0.878	-1.802	0.875
Parabacteroides	-1.383	0.813	-1.701	0.089	0.666	-2.976	0.210
Bacillus	0.325	0.490	0.662	0.508	0.878	-0.636	1.286
Coprobacillus	0.165	0.443	0.371	0.710	0.974	-0.705	1.034
Erysipelatoclostridium	0.413	0.453	0.912	0.362	0.791	-0.474	1.300
Faecalitalea	0.234	0.530	0.441	0.659	0.959	-0.804	1.272
Holdemania	-0.230	0.176	-1.305	0.192	0.791	-0.576	0.116
Turicibacter	-0.347	0.472	-0.736	0.462	0.865	-1.272	0.577
Granulicatella	0.181	0.385	0.471	0.637	0.949	-0.573	0.935
Enterococcus	0.258	0.456	0.565	0.572	0.915	-0.637	1.153
Lactobacillus	-0.054	0.428	-0.126	0.900	0.990	-0.893	0.786
Streptococcus	-0.011	0.316	-0.034	0.973	0.990	-0.630	0.608
Clostridium	0.909	0.448	2.029	**0.042***	0.452	0.031	1.786
Intestinibacter	-0.021	0.472	-0.044	0.965	0.990	-0.945	0.904
Romboutsia	-0.450	0.410	-1.097	0.273	0.791	-1.254	0.354
Agathobacter	-0.448	0.624	-0.718	0.473	0.865	-1.672	0.776
Anaerostipes	-0.427	0.352	-1.215	0.224	0.791	-1.116	0.262
Blautia	-0.376	0.499	-0.753	0.451	0.865	-1.354	0.602
Dorea	0.109	0.549	0.199	0.842	0.980	-0.967	1.185
Eisenbergiella	0.127	0.512	0.248	0.804	0.979	-0.877	1.131
Fusicatenibacter	0.772	0.554	1.393	0.164	0.748	-0.314	1.859
Hungatella	0.039	0.414	0.094	0.925	0.990	-0.773	0.850
Lachnoclostridium	0.523	0.216	2.423	**0.015***	0.452	0.100	0.946
Lachnospira	-0.044	0.480	-0.092	0.927	0.990	-0.986	0.897
Roseburia	-0.458	0.547	-0.836	0.403	0.832	-1.530	0.615
Sellimonas	0.018	0.437	0.042	0.967	0.990	-0.839	0.875
Tuzzerella	0.102	0.358	0.285	0.776	0.974	-0.599	0.803
Tyzzerella	1.106	0.651	1.698	0.089	0.666	-0.170	2.382

Lachnospiraceae_UCG-001	0.761	0.332	2.295	**0.022***	0.452	0.111	1.411
Lachnospiraceae_UCG-004	-0.181	0.512	-0.352	0.725	0.974	-1.185	0.824
Colidextribacter	0.005	0.399	0.013	0.990	0.990	-0.776	0.786
Flavonifractor	0.496	0.237	2.097	**0.036***	0.452	0.032	0.960
Oscillibacter	0.217	0.378	0.574	0.566	0.915	-0.524	0.958
Oscillospira	-0.078	0.272	-0.287	0.774	0.974	-0.612	0.455
Anaerotruncus	-0.167	0.206	-0.813	0.416	0.833	-0.571	0.236
Butyricicoccus	-0.422	0.373	-1.134	0.257	0.791	-1.153	0.308
Candidatus Soleaferrea	0.133	0.250	0.529	0.596	0.931	-0.358	0.623
Faecalibacterium	0.119	0.400	0.297	0.767	0.974	-0.665	0.902
Negativibacillus	0.005	0.328	0.016	0.987	0.990	-0.639	0.649
Ruminococcus	-0.056	0.738	-0.076	0.940	0.990	-1.502	1.390
Subdoligranulum	-0.632	0.547	-1.155	0.248	0.791	-1.704	0.441
UBA1819	-0.498	0.333	-1.498	0.134	0.716	-1.150	0.154
Monoglobus	-1.016	0.495	-2.050	**0.040***	0.452	-1.987	-0.045
Dialister	0.523	0.548	0.953	0.340	0.791	-0.552	1.597
Veillonella	0.710	0.614	1.156	0.248	0.791	-0.493	1.913
Bilophila	-0.140	0.485	-0.288	0.773	0.974	-1.091	0.811
Parasutterella	-0.990	0.591	-1.676	0.094	0.666	-2.148	0.168
Sutterella	-0.781	0.737	-1.059	0.290	0.791	-2.225	0.664
Escherichia-Shigella	0.902	0.596	1.514	0.130	0.716	-0.266	2.070
Haemophilus	0.120	0.543	0.220	0.826	0.979	-0.945	1.184
Akkermansia	-0.079	0.329	-0.239	0.811	0.979	-0.723	0.566

Note. Antibiotic treatment within the last 3 months and sex were used as covariates.
* $p < 0.05$; beta, coefficient obtained from the ANCOM-BC log-linear model; se, standard error of beta; W, test statistic (beta/se)

Table S12. *Demographic details between the EM-risk and EM non-risk groups*

**Items**	**All participants**	**EM-risk group**	**EM non-risk group**	***p*-value**	***q-value***
Sample (N)	257	20	237		
Antibiotics treatment within the last 3 months (N)	32	3	29		
Age (months) [a]	46.53 (6.33)	45.55 (6.40)	46.62 (6.33)	0.47	0.81
Sex (boy/girl)	152/105	14/6	138/99	0.35	0.81
BMI [a]	15.76 (1.29)	16.23 (2.39)	15.72 (1.16)	0.94	0.94
Family income [b]	less than 3 million yen: 21 (8%)<br>3-5 million yen: 71 (30%)<br>5-7 million yen: 80 (34%)<br>more than 7 million yen: 65 (27%)<br>Not answered: 20	less than 3 million yen: 2 (12%)<br>3-5 million yen: 5 (29%)<br>5-7 million yen: 7 (41%)<br>more than 7 million yen: 3 (18%)<br>Not answered: 3	less than 3 million yen: 19 (9%)<br>3-5 million yen: 66 (30%)<br>5-7 million yen: 73 (33%)<br>more than 7 million yen: 62 (28%)<br>Not answered: 17	0.74	0.87
Mother's years of schooling [a]	14.77 (1.88)	14.70 (2.00)	14.77 (1.87)	0.53	0.81
Siblings [b]	Yes: 210 (82%)<br>No: 45 (18%)<br>Not answered: 2	Yes: 15 (75%)<br>No: 5 (25%)<br>Not answered: 0	Yes: 195 (83%)<br>No: 40 (17%)<br>Not answered: 2	0.36	0.81
Birth mode [b]	Vaginal delivery: 201 (78%)<br>C-section: 56 (22%)	Vaginal delivery: 17 (85%)<br>C-section: 3 (15%)	Vaginal delivery: 184 (78%)<br>C-section: 53 (22%)	0.58	0.81

[a]Mean (SD). [b] N (%)

Table S13. *Comparison of diversity of the microbiota between EM-risk (N = 20) and EM non-risk (N = 237) groups (ANCOVA, PERMANOVA)*

Alpha diversity	**All analyzed data**	**EM-risk group**	**EM non-risk group**	***F***	***p*-value**	***q*-value**	***η2G***
Shannon [a]	5.83 (0.49)	5.99 (0.56)	5.82 (0.49)	2.547	0.11	0.75	0.010
Chao1 [a]	169.75 (44.07)	176.96 (50.20)	169.14 (43.57)	0.586	0.45	0.96	0.002
Faith's PD [a]	22.42 (4.88)	22.57 (4.52)	22.41 (4.92)	0.024	0.88	0.98	9.56e-05
Observed species [a]	169.54 (44.04)	168.93 (43.53)	176.8 (50.34)	0.597	0.44	0.96	0.002
Beta diversity				***F***	***p*-value**	***q*-value**	***R*2**
EM risk vs. EM non-risk				0.858	0.56	0.96	0.003

Note. Antibiotic treatment within the last 3 months and sex were used as covariates.
[a]Mean (SD)

Table S14. *Comparison of relative abundance of the microbiota between EM-risk (N = 20) and EM non-risk (N = 237) groups (ANCOM-BC)*

Genus	beta	SE	W	*p-value*	*q-value*	95% CI	
						low	high
Actinomyces	0.438	0.367	1.195	0.232	0.959	-0.280	1.157
Bifidobacterium	0.018	0.184	0.098	0.922	0.984	-0.342	0.378
Collinsella	1.244	0.680	1.830	0.067	0.754	-0.089	2.577
Eggerthella	-0.192	0.248	-0.774	0.439	0.960	-0.679	0.295
Gordonibacter	-0.218	0.194	-1.124	0.261	0.959	-0.597	0.162
Bacteroides	0.079	0.111	0.711	0.477	0.960	-0.139	0.297
Odoribacter	-0.278	0.490	-0.568	0.570	0.960	-1.239	0.683
Prevotella	-0.386	0.519	-0.744	0.457	0.960	-1.402	0.631
Alistipes	0.223	0.530	0.421	0.674	0.963	-0.816	1.263
Parabacteroides	1.005	0.566	1.775	0.076	0.754	-0.105	2.116
Bacillus	0.190	0.458	0.414	0.679	0.963	-0.708	1.087
Coprobacillus	0.123	0.358	0.342	0.732	0.984	-0.579	0.825
Erysipelatoclostridium	0.431	0.427	1.009	0.313	0.960	-0.406	1.268
Faecalitalea	-0.659	0.401	-1.643	0.100	0.754	-1.446	0.127
Holdemania	-0.386	0.133	-2.903	**0.004****	0.237	-0.647	-0.126
Turicibacter	-0.374	0.421	-0.888	0.375	0.960	-1.198	0.451
Granulicatella	0.069	0.420	0.165	0.869	0.984	-0.754	0.892
Enterococcus	-0.029	0.374	-0.078	0.938	0.984	-0.762	0.704
Lactobacillus	0.293	0.479	0.613	0.540	0.960	-0.645	1.232
Streptococcus	-0.037	0.322	-0.115	0.909	0.984	-0.667	0.593
Clostridium	-0.128	0.420	-0.306	0.760	0.984	-0.951	0.694
Intestinibacter	0.046	0.421	0.110	0.912	0.984	-0.779	0.872
Romboutsia	-0.587	0.388	-1.516	0.130	0.754	-1.347	0.172
Agathobacter	-0.012	0.664	-0.018	0.986	1.000	-1.314	1.290
Anaerostipes	0.058	0.287	0.201	0.841	0.984	-0.504	0.619
Blautia	0.215	0.372	0.576	0.565	0.960	-0.515	0.944
Dorea	0.806	0.478	1.686	0.092	0.754	-0.131	1.744
Eisenbergiella	-0.346	0.341	-1.016	0.310	0.960	-1.013	0.321
Fusicatenibacter	-0.465	0.676	-0.687	0.492	0.960	-1.790	0.861
Hungatella	0.241	0.358	0.673	0.501	0.960	-0.461	0.943
Lachnoclostridium	0.095	0.230	0.414	0.679	0.963	-0.355	0.545
Lachnospira	0.000	0.493	0.000	1.000	1.000	-0.966	0.967
Roseburia	-0.255	0.529	-0.483	0.629	0.963	-1.291	0.781
Sellimonas	0.222	0.369	0.600	0.548	0.960	-0.502	0.945
Tuzzerella	-0.506	0.326	-1.551	0.121	0.754	-1.146	0.133
Tyzzerella	0.117	0.509	0.231	0.818	0.984	-0.880	1.114

Lachnospiraceae_UCG-001	0.652	0.388	1.680	0.093	0.754	-0.109	1.414
Lachnospiraceae_UCG-004	0.037	0.453	0.081	0.935	0.984	-0.851	0.925
Colidextribacter	-0.162	0.401	-0.404	0.686	0.963	-0.949	0.624
Flavonifractor	-0.084	0.273	-0.306	0.760	0.984	-0.618	0.451
Oscillibacter	0.008	0.369	0.021	0.983	1.000	-0.715	0.731
Oscillospira	-0.311	0.252	-1.235	0.217	0.959	-0.805	0.182
Anaerotruncus	-0.180	0.222	-0.809	0.418	0.960	-0.616	0.256
Butyricicoccus	-0.423	0.376	-1.127	0.260	0.959	-1.160	0.313
Candidatus Soleaferrea	0.078	0.153	0.510	0.610	0.963	-0.221	0.377
Faecalibacterium	0.101	0.391	0.258	0.796	0.984	-0.665	0.867
Negativibacillus	-0.380	0.187	-2.031	**0.042***	0.754	-0.746	-0.013
Ruminococcus	0.273	0.643	0.425	0.671	0.963	-0.987	1.534
Subdoligranulum	0.062	0.531	0.118	0.906	0.984	-0.978	1.103
UBA1819	-0.030	0.320	-0.094	0.925	0.984	-0.657	0.597
Monoglobus	-0.612	0.482	-1.269	0.205	0.959	-1.557	0.333
Dialister	-0.670	0.463	-1.447	0.148	0.789	-1.578	0.238
Veillonella	0.587	0.634	0.926	0.354	0.960	-0.656	1.831
Bilophila	-0.305	0.415	-0.734	0.463	0.960	-1.119	0.509
Parasutterella	-0.458	0.536	-0.855	0.393	0.960	-1.509	0.592
Sutterella	1.186	0.622	1.906	0.057	0.754	-0.033	2.405
Escherichia-Shigella	-0.321	0.515	-0.622	0.534	0.960	-1.330	0.689
Haemophilus	0.679	0.615	1.104	0.270	0.959	-0.527	1.886
Akkermansia	-0.113	0.284	-0.396	0.692	0.963	-0.670	0.445

Note. Antibiotic treatment within the last 3 months and sex were used as covariates.
** $p < 0.01$; * $p < 0.05$; beta, coefficient obtained from the ANCOM-BC log-linear model; se, standard error of beta; W, test statistic (beta/se)

Table S15. *Demographic details compared between ISC-risk and ISC-control groups (excluding participants in FL-risk or EM-risk group)*

**Variable**	**All analyzed data**	**ISC-risk group**	**ISC-control group**	***p*-value**	***q*-value**
Sample (N)	242	26	216		
Antibiotic treatment within the last 3 months (N)	30	3 (12%)	27 (13%)		
Age (months) [a]	46.61 (6.31)	46.04 (6.32)	46.68 (6.32)	0.67	0.91
Sex (boy/girl)	141/101	19/7	122/94	0.14	0.49
BMI [a]	15.73 (1.26)	16.43 (1.89)	15.66 (1.16)	0.06	0.44
Family income [b]	less than 3 million yen: 19 (9%)<br>3-5 million yen: 65 (29%)<br>5-7 million yen: 78 (35%)<br>more than 7 million yen: 62 (28%)<br>Not answered: 18	less than 3 million yen: 1 (4%)<br>3-5 million yen: 8 (33%)<br>5-7 million yen: 11 (46%)<br>more than 7 million yen: 4 (17%)<br>Not answered: 2	less than 3 million yen: 18 (9%)<br>3-5 million yen: 57 (29%)<br>5-7 million yen: 67 (33%)<br>more than 7 million yen: 58 (29%)<br>Not answered: 16	0.48	0.83
Mother's years of schooling [a]	14.78 (1.88)	14.50 (2.08)	14.82 (1.85)	0.26	0.60
Siblings [b]	Yes: 200 (83%)<br>No: 40 (17%)<br>Not answered: 2	Yes: 21 (81%)<br>No: 5 (19%)<br>Not answered: 0	Yes: 179 (84%)<br>No: 35 (16%)<br>Not answered: 2	0.78	0.91
Birth mode [b]	Vaginal delivery: 188 (78%)<br>C-section: 54 (22%)	Vaginal delivery: 20 (77%)<br>C-section: 6 (23%)	Vaginal delivery: 168 (78%)<br>C-section: 48 (22%)	1.00	1.00

Note. Participants exceeding the cutoff values for either *Flexibility* and/or *Emergent Metacognition* on the BRIEF-P were excluded from the ISC-control group.
[a]Mean (SD). [b] N (%)

Table S16. *Comparison of diversity of the microbiota between ISC-risk (N = 26) and ISC-control (N =216) groups (excluding participants in the FL-risk or EM-risk group) (ANCOVA, PERMANOVA)*

Alpha diversity	**All analyzed data**	**ISC-risk group**	**ISC-control group**	***F***	***p*-value**	***q*-value**	***η2G***
Shannon [a]	5.82 (0.49)	5.82 (0.48)	5.85 (0.61)	0.133	0.72	0.99	0.0006
Chao1 [a]	169.74 (44.09)	172.63 (50.82)	169.4 (43.33)	0.087	0.77	0.99	0.0004
Faith's PD [a]	22.46 (4.92)	22.74 (5.08)	22.42 (4.91)	0.071	0.79	0.99	0.0003
Observed species [a]	169.56 (44.05)	172.54 (50.88)	169.20 (43.27)	0.094	0.76	0.99	0.0004
Beta diversity				***F***	***p*-value**	***q*-value**	***R2***
ISC-risk vs. ISC-control				0.681	0.77	0.99	0.0028

Note. Participants exceeding the cutoff values for either Flexibility or Emergent Metacognition on the BRIEF-P were excluded from the ISC-control group. Antibiotic treatment within the last 3 months and sex were used as covariates.

[a]Mean (SD)

Table S17. *Comparison of relative abundance of the microbiota between ISC-risk (N = 26) and ISC-control (N =216) groups (Excluding participants in the FL-risk and/or EM-risk group) (ANCOM-BC)*

**Genus**	**beta**	**SE**	**W**	***p*-value**	***q*-value**	**95% CI**	
						**low**	**high**
Actinomyces	0.96	0.25	3.78	**0.0002*** **	**0.010***	0.46	1.46
Bifidobacterium	0.04	0.15	0.27	0.7887	0.987	-0.25	0.32
Collinsella	0.42	0.60	0.71	0.4798	0.987	-0.75	1.59
Eggerthella	-0.04	0.21	-0.17	0.8670	0.987	-0.45	0.38
Gordonibacter	0.08	0.20	0.39	0.6940	0.987	-0.31	0.47
Bacteroides	0.08	0.11	0.70	0.4828	0.987	-0.14	0.29
Odoribacter	0.52	0.45	1.16	0.2472	0.931	-0.36	1.41
Prevotella	0.07	0.46	0.16	0.8729	0.987	-0.82	0.97
Alistipes	-0.08	0.55	-0.15	0.8813	0.987	-1.16	0.99
Parabacteroides	0.05	0.59	0.09	0.9275	0.989	-1.10	1.21
Bacillus	0.60	0.41	1.46	0.1436	0.707	-0.20	1.41
Coprobacillus	0.13	0.39	0.34	0.7351	0.987	-0.63	0.90
Erysipelatoclostridium	0.29	0.37	0.78	0.4352	0.987	-0.44	1.01
Faecalitalea	-0.19	0.38	-0.49	0.6258	0.987	-0.93	0.56
Holdemania	-0.26	0.15	-1.71	0.0869	0.681	-0.56	0.04
Turicibacter	-0.02	0.39	-0.06	0.9494	0.996	-0.79	0.74
Granulicatella	0.35	0.34	1.05	0.2933	0.987	-0.31	1.01
Enterococcus	0.45	0.36	1.24	0.2163	0.923	-0.26	1.16
Lactobacillus	-0.34	0.40	-0.84	0.3990	0.987	-1.12	0.44
Streptococcus	0.11	0.29	0.37	0.7129	0.987	-0.47	0.68
Clostridium	0.50	0.42	1.19	0.2343	0.931	-0.33	1.33
Intestinibacter	-0.01	0.36	-0.02	0.9841	1.000	-0.72	0.70
Romboutsia	0.18	0.36	0.48	0.6281	0.987	-0.53	0.89
Agathobacter	0.07	0.58	0.12	0.9043	0.987	-1.06	1.20
Anaerostipes	-0.15	0.30	-0.49	0.6240	0.987	-0.74	0.44
Blautia	-0.63	0.42	-1.52	0.1278	0.681	-1.45	0.18
Dorea	0.38	0.43	0.89	0.3729	0.987	-0.46	1.22
Eisenbergiella	0.04	0.38	0.11	0.9095	0.987	-0.69	0.78
Fusicatenibacter	0.51	0.45	1.12	0.2624	0.933	-0.38	1.40
Hungatella	0.24	0.33	0.72	0.4716	0.987	-0.41	0.89
Lachnoclostridium	0.27	0.21	1.31	0.1897	0.867	-0.14	0.68
Lachnospira	-0.81	0.42	-1.93	0.0539	0.681	-1.62	0.01
Roseburia	-0.78	0.43	-1.80	0.0723	0.681	-1.62	0.07
Sellimonas	-0.01	0.35	-0.03	0.9728	0.987	-0.69	0.67
Tuzzerella	-0.48	0.30	-1.62	0.1053	0.681	-1.07	0.10
Tyzzerella	0.15	0.48	0.30	0.7648	1.000	-0.81	1.10

Lachnospiraceae_UCG-001	0.47	0.30	1.59	0.1122	0.681	-0.11	1.06
Lachnospiraceae_UCG-004	-0.18	0.40	-0.46	0.6477	0.987	-0.97	0.60
Colidextribacter	0.14	0.36	0.38	0.7021	0.987	-0.56	0.83
Flavonifractor	0.00	0.26	0.00	0.9998	1.000	-0.51	0.51
Oscillibacter	0.18	0.34	0.52	0.6025	0.987	-0.49	0.85
Oscillospira	-0.16	0.26	-0.61	0.5413	0.987	-0.68	0.35
Anaerotruncus	-0.04	0.17	-0.24	0.8069	0.987	-0.38	0.29
Butyricicoccus	-0.58	0.34	-1.69	0.0911	0.681	-1.26	0.09
Candidatus Soleaferrea	0.21	0.22	0.95	0.3411	0.987	-0.22	0.63
Faecalibacterium	0.13	0.30	0.44	0.6611	0.987	-0.45	0.72
Negativibacillus	-0.13	0.25	-0.52	0.6047	0.987	-0.61	0.36
Ruminococcus	-0.41	0.57	-0.72	0.4733	0.987	-1.53	0.71
Subdoligranulum	-0.39	0.45	-0.87	0.3850	0.987	-1.27	0.49
UBA1819	-0.53	0.32	-1.67	0.0958	0.681	-1.15	0.09
Monoglobus	-0.93	0.45	-2.08	**0.0375***	0.681	-1.80	-0.05
Dialister	-0.13	0.41	-0.32	0.7518	0.987	-0.92	0.67
Veillonella	0.18	0.59	0.31	0.7544	0.987	-0.97	1.34
Bilophila	0.06	0.39	0.16	0.8702	0.987	-0.70	0.83
Parasutterella	-0.74	0.48	-1.53	0.1259	0.681	-1.68	0.21
Sutterella	1.55	0.52	2.97	**0.0029****	**0.094†**	0.53	2.57
Escherichia-Shigella	-0.22	0.55	-0.40	0.6872	0.987	-1.31	0.86
Haemophilus	-0.09	0.48	-0.19	0.8530	0.987	-1.02	0.85
Akkermansia	0.15	0.28	0.55	0.5847	0.987	-0.39	0.70

Note. Participants exceeding the cutoff values for either Emergent metacognition or Flexibility on the BRIEF-P were excluded from the ISC-control group. Antibiotic treatment within the last 3 months and sex were used as covariates.
*** $p < 0.001$; ** p or $q < 0.01$; * $p < 0.05$; †$q < 0.10$; beta, coefficient obtained from the ANCOM-BC log-linear model; se, standard error of beta; W, test statistic (beta/se)

Table S18. *Demographic details between FL-risk and FL-control groups (excluding participants in the ISC-risk or EM-risk group)*

**Variable**	**All analyzed data**	**FL-risk group**	**FL-control group**	***p*-value**	***q*-value**
Sample (N)	234	18	216		
Antibiotic treatment within the last 3 months (N)	30	3	27		
Age (months) [a]	46.52 (6.34)	44.56 (6.46)	46.68 (6.32)	0.174	0.243
Sex (boy/girl)	136/100	15/3	122/94	0.027*	0.094†
BMI [a]	15.71 (1.17)	16.35 (1.18)	15.66 (1.16)	0.049*	0.114
Family income [b]	less than 3 million yen: 18 (8%)<br>3-5 million yen: 57 (26%)<br>5-7 million yen: 67 (31%)<br>more than 7 million yen: 58 (27%)<br>Not answered: 18	less than 3 million yen: 0 (0%)<br>3-5 million yen: 9 (56%)<br>5-7 million yen: 5 (31%)<br>more than 7 million yen: 2 (13%)<br>Not answered: 2	less than 3 million yen: 18 (9%)<br>3-5 million yen: 57 (29%)<br>5-7 million yen: 67 (34%)<br>more than 7 million yen: 58 (29%)<br>Not answered: 16	0.119	0.207
Mother's years of schooling [a]	14.81 (1.90)	14.72 (2.40)	14.82 (1.85)	0.419	0.488
Siblings [b]	Yes: 189 (81%)<br>No: 43 (19%)<br>Not answered: 2	Yes: 10 (56%)<br>No: 8 (44%)<br>Not answered: 0	Yes: 179 (83%)<br>No: 35 (17%)<br>Not answered: 2	0.008**	0.053†
Birth mode [b]	Vaginal delivery: 183 (78%)<br>C-section: 51 (22%)	Vaginal delivery: 15 (83%)<br>C-section: 3 (17%)	Vaginal delivery: 168 (78%)<br>C-section: 48 (22%)	0.770	0.770

Note. Participants exceeding the cutoff values for either *Inhibitory Self-Control* or *Emergent Metacognition* on the BRIEF-P were excluded from the FL-control group.
[a] Mean (SD). [b] N (%)
** $p < 0.01$, * $p < 0.05$, † $q < 0.10$

Table S19. *Comparison of diversity of the microbiota between FL-risk (N = 18) and FL-control (N = 216) groups (excluding participants in the ISC-risk or EM-risk group) (ANCOVA, PERMANOVA)*

Alpha diversity	**All analyzed data**	**FL-risk group**	**FL-control group**	***F***	***p*-value**	***q*-value**	***η2G***
Shannon [a]	5.82 (0.48)	5.76 (0.56)	5.82 (0.48)	0.090	0.76	0.85	0.0003
Chao1 [a]	168.74 (43.31)	160.91 (43.48)	169.40 (43.33)	0.541	0.46	0.84	0.0020
Faith's PD [a]	22.37 (4.86)	21.76 (4.34)	22.42 (4.91)	0.242	0.62	0.84	0.0010
Observed species [a]	168.53 (43.26)	160.50 (43.52)	169.20 (43.27)	0.567	0.45	0.84	0.0020
Beta diversity				***F***	***p*-value**	***q*-value**	***R*2**
FL-risk vs. FL-control				0.681	0.12	0.59	0.0028

Note. Participants exceeding the cutoff values for either *Inhibitory Self-Control* or *Emergent Metacognition* on the BRIEF-P were excluded from the FL-control group. Antibiotic treatment within the last 3 months and sex were used as covariates.
[a]Mean (SD)

Table S20. *Comparison of relative abundance of the microbiota between FL-risk (N = 18) and FL-control (N = 216) groups (excluding participants in the ISC-risk or EM-risk group) (ANCOM-BC)*

Genus	beta	SE	W	*p*-value	*q*-value	95% CI	
						low	high
Actinomyces	0.590	0.303	1.945	0.052	0.494	-0.005	1.185
Bifidobacterium	-0.054	0.167	-0.324	0.746	0.850	-0.382	0.273
Collinsella	-0.384	0.665	-0.578	0.563	0.838	-1.687	0.919
Eggerthella	0.154	0.197	0.780	0.435	0.838	-0.232	0.540
Gordonibacter	-0.341	0.206	-1.650	0.099	0.593	-0.745	0.064
Bacteroides	0.239	0.127	1.882	0.060	0.494	-0.010	0.487
Odoribacter	0.535	0.513	1.042	0.297	0.838	-0.471	1.541
Prevotella	-0.330	0.492	-0.672	0.502	0.838	-1.294	0.634
Alistipes	-0.375	0.681	-0.551	0.582	0.838	-1.710	0.960
Parabacteroides	-1.151	0.817	-1.408	0.159	0.671	-2.753	0.451
Coprobacillus	0.253	0.445	0.569	0.569	0.838	-0.619	1.126
Erysipelatoclostridium	0.503	0.456	1.104	0.270	0.838	-0.390	1.397
Faecalitalea	0.265	0.529	0.501	0.617	0.838	-0.771	1.300
Holdemania	-0.185	0.181	-1.025	0.305	0.838	-0.539	0.169
Turicibacter	-0.220	0.471	-0.468	0.640	0.841	-1.144	0.703
Granulicatella	0.278	0.385	0.722	0.470	0.838	-0.477	1.034
Enterococcus	0.370	0.462	0.801	0.423	0.838	-0.536	1.276
Lactobacillus	0.001	0.433	0.003	0.997	0.917	-0.848	0.851
Streptococcus	0.069	0.320	0.215	0.830	0.872	-0.559	0.696
Clostridium	0.988	0.453	2.181	**0.029***	0.422	0.100	1.876
Intestinibacter	0.042	0.475	0.089	0.929	0.896	-0.888	0.973
Romboutsia	-0.341	0.414	-0.824	0.410	0.838	-1.152	0.470
Agathobacter	-0.347	0.626	-0.555	0.579	0.838	-1.575	0.880
Anaerostipes	-0.299	0.355	-0.844	0.399	0.838	-0.995	0.396
Blautia	-0.267	0.498	-0.536	0.592	0.838	-1.244	0.709
Dorea	0.341	0.549	0.622	0.534	0.838	-0.735	1.417
Eisenbergiella	0.192	0.516	0.372	0.710	0.850	-0.819	1.203
Fusicatenibacter	0.872	0.559	1.560	0.119	0.593	-0.224	1.968
Hungatella	0.163	0.419	0.390	0.696	0.850	-0.657	0.984
Lachnoclostridium	0.601	0.219	2.736	**0.006****	0.188	0.170	1.031
Lachnospira	0.024	0.485	0.050	0.960	0.910	-0.927	0.975
Roseburia	-0.416	0.550	-0.757	0.449	0.838	-1.495	0.662
Sellimonas	0.112	0.435	0.258	0.796	0.853	-0.740	0.965
Tuzzerella	0.117	0.357	0.327	0.744	0.850	-0.583	0.816
Tyzzerella	1.123	0.654	1.717	0.086	0.593	-0.159	2.404
Lachnospiraceae_UCG-001	0.908	0.333	2.722	**0.006****	0.188	0.254	1.561

Lachnospiraceae_UCG-004	-0.079	0.516	-0.153	0.878	0.896	-1.090	0.932
Colidextribacter	0.124	0.399	0.311	0.756	0.850	-0.657	0.905
Flavonifractor	0.569	0.240	2.370	**0.018***	0.343	0.099	1.040
Oscillibacter	0.321	0.377	0.851	0.395	0.838	-0.419	1.061
Oscillospira	0.000	0.271	0.001	0.999	0.917	-0.531	0.532
Anaerotruncus	-0.074	0.204	-0.362	0.717	0.850	-0.474	0.326
Butyricicoccus	-0.393	0.371	-1.060	0.289	0.838	-1.120	0.334
Candidatus Soleaferrea	0.239	0.250	0.953	0.340	0.838	-0.252	0.729
Faecalibacterium	0.200	0.399	0.501	0.616	0.838	-0.582	0.982
Negativibacillus	0.087	0.331	0.263	0.793	0.853	-0.561	0.735
Ruminococcus	0.079	0.742	0.107	0.915	0.896	-1.374	1.533
Subdoligranulum	-0.513	0.548	-0.937	0.349	0.838	-1.586	0.560
UBA1819	-0.453	0.333	-1.359	0.174	0.671	-1.107	0.200
Monoglobus	-0.950	0.499	-1.906	0.057	0.494	-1.928	0.027
Dialister	0.538	0.554	0.970	0.332	0.838	-0.548	1.624
Veillonella	0.763	0.619	1.233	0.218	0.786	-0.450	1.977
Bilophila	-0.052	0.490	-0.106	0.916	0.896	-1.013	0.909
Parasutterella	-0.953	0.591	-1.613	0.107	0.593	-2.112	0.205
Sutterella	-0.448	0.743	-0.603	0.547	0.838	-1.904	1.009
Escherichia-Shigella	0.835	0.605	1.381	0.167	0.671	-0.350	2.020
Haemophilus	0.202	0.545	0.371	0.711	0.850	-0.866	1.271
Akkermansia	0.039	0.330	0.120	0.905	0.896	-0.607	0.686

Note. Participants exceeding the cutoff values for either *Inhibitory Self-Control* or *Emergent Metacognition* on the BRIEF-P were excluded from the FL-control group. Antibiotic treatment within the last 3 months and sex were used as covariates.
* $p < 0.05$; beta, coefficient obtained from the ANCOM-BC log-linear model; se, standard error of beta; W, test statistic (beta/se)

Table S21. *Demographic details between the EM-risk and EM-control groups (excluding participants in the ISC-risk or FL-risk group)*

**Variable**	**All analyzed data**	**EM-risk group**	**EM-control group**	***p*-value**	***q*-value**
Sample (N)	236	20	216		
Antibiotic treatment within the last 3 months (N)	30	3	27		
Age (months) [a]	46.58 (6.32)	45.55 (6.40)	46.68 (6.32)	0.44	0.78
Sex (boy/girl)	136/100	14/6	122/94	0.34	0.78
BMI [a]	15.70 (1.30)	16.23 (2.39)	15.66 (1.16)	0.83	0.83
Family income [b]	less than 3 million yen: 20 (9%)<br>3-5 million yen: 62 (29%)<br>5-7 million yen: 74 (34%)<br>more than 7 million yen: 61 (28%)<br>Not answered: 19	less than 3 million yen: 2 (12%)<br>3-5 million yen: 5 (29%)<br>5-7 million yen: 7 (41%)<br>more than 7 million yen: 3 (18%)<br>Not answered: 3	less than 3 million yen: 18 (9%)<br>3-5 million yen: 57 (29%)<br>5-7 million yen: 67 (34%)<br>more than 7 million yen: 58 (29%)<br>Not answered: 16	0.77	0.83
Mother's years of schooling [a]	14.81 (1.86)	14.70 (2.00)	14.82 (1.85)	0.45	0.78
Siblings [b]	Yes: 200 (83%)<br>No: 40 (17%)<br>Not answered: 2	Yes: 15 (75%)<br>No: 5 (25%)<br>Not answered: 0	Yes: 179 (83%)<br>No: 35 (17%)<br>Not answered: 4	0.35	0.78
Birth mode [b]	Vaginal delivery: 185 (78%)<br>C-section: 51 (22%)	Vaginal delivery: 17 (85%)<br>C-section: 3 (15%)	Vaginal delivery: 168 (78%)<br>C-section: 48 (22%)	0.58	0.81

Note. Participants exceeding the cutoff values for either *Inhibitory Self-Control* or *Flexibility* on the BRIEF-P were excluded from the EM-control group.
[a]Mean (SD). [b] N (%)

Table S22. *Comparison of diversity of the microbiota between EM-risk (N = 20) and EM-control (N = 216) groups (excluding participants in the ISC-risk or FL-risk group) (ANCOVA, PERMANOVA)*

Alpha diversity	**All analyzed data**	**EM-risk group**	**EM-control group**	***F***	***p*-value**	***q*-value**	***η2G***
Shannon [a]	5.84 (0.49)	5.99 (0.56)	5.82 (0.48)	2.578	0.11	0.75	0.0110
Chao1 [a]	170.04 (43.89)	176.96 (50.20)	169.40 (43.33)	0.597	0.44	0.97	0.0030
Faith's PD [a]	22.44 (4.87)	22.57 (4.52)	22.42 (4.91)	0.031	0.86	0.99	0.0001
Observed species [a]	169.85 (43.85)	176.80 (50.34)	169.20 (43.27)	0.605	0.44	0.97	0.0030
Beta diversity				***F***	***p*-value**	***q*-value**	***R*2**
EM-risk vs. EM-control				0.861	0.56	0.98	0.0037

Note. Participants exceeding the cutoff values for either *Inhibitory Self-Control* or *Flexibility* on the BRIEF-P were excluded from the EM-control group. Antibiotic treatment within the last 3 months and sex were used as covariates.
[a]Mean (SD)

Table S23. *Comparison of relative abundance of the microbiota between EM-risk (N = 20) and EM-control (N = 216) groups (excluding participants in the ISC-risk or FL-risk group) (ANCOM-BC)*

**Genus**	**beta**	**SE**	**W**	***p*-value**	***q*-value**	**95% CI**	
						**low**	**high**
Actinomyces	0.490	0.366	1.340	0.180	0.811	-0.227	1.207
Bifidobacterium	0.006	0.187	0.035	0.972	0.991	-0.359	0.372
Collinsella	1.213	0.683	1.774	0.076	0.753	-0.127	2.552
Eggerthella	-0.182	0.251	-0.724	0.469	0.973	-0.675	0.311
Gordonibacter	-0.215	0.193	-1.119	0.263	0.948	-0.593	0.162
Bacteroides	0.078	0.110	0.709	0.479	0.973	-0.138	0.295
Odoribacter	-0.198	0.483	-0.410	0.682	0.991	-1.146	0.749
Prevotella	-0.380	0.520	-0.730	0.465	0.973	-1.399	0.640
Alistipes	0.172	0.525	0.328	0.743	0.991	-0.856	1.200
Parabacteroides	0.924	0.573	1.611	0.107	0.753	-0.200	2.048
Coprobacillus	0.141	0.357	0.395	0.693	0.991	-0.559	0.840
Erysipelatoclostridium	0.432	0.425	1.018	0.309	0.948	-0.400	1.265
Faecalitalea	-0.627	0.403	-1.556	0.120	0.753	-1.417	0.163
Holdemania	-0.409	0.136	-3.017	**0.003****	0.161	-0.675	-0.143
Turicibacter	-0.376	0.422	-0.890	0.373	0.973	-1.202	0.451
Granulicatella	0.085	0.418	0.202	0.840	0.991	-0.735	0.905
Enterococcus	0.004	0.372	0.012	0.991	0.991	-0.725	0.734
Lactobacillus	0.231	0.484	0.477	0.633	0.991	-0.717	1.179
Streptococcus	-0.043	0.321	-0.135	0.893	0.991	-0.673	0.586
Clostridium	-0.070	0.418	-0.167	0.867	0.991	-0.889	0.750
Intestinibacter	0.016	0.421	0.037	0.970	0.991	-0.810	0.841
Romboutsia	-0.580	0.388	-1.495	0.135	0.773	-1.341	0.181
Agathobacter	-0.032	0.665	-0.048	0.962	0.991	-1.335	1.271
Anaerostipes	0.046	0.286	0.162	0.872	0.991	-0.515	0.607
Blautia	0.170	0.374	0.454	0.650	0.991	-0.564	0.903
Dorea	0.860	0.484	1.775	0.076	0.753	-0.089	1.809
Eisenbergiella	-0.343	0.336	-1.021	0.307	0.948	-1.003	0.316
Fusicatenibacter	-0.371	0.679	-0.546	0.585	0.991	-1.702	0.960
Hungatella	0.254	0.356	0.714	0.475	0.973	-0.443	0.952
Lachnoclostridium	0.112	0.226	0.497	0.620	0.991	-0.331	0.556
Lachnospira	-0.054	0.497	-0.108	0.914	0.991	-1.029	0.921
Roseburia	0.686	0.394	1.739	0.082	0.753	-0.087	1.459
Sellimonas	0.040	0.454	0.088	0.930	0.991	-0.850	0.930
Tuzzerella	-0.314	0.527	-0.595	0.552	0.982	-1.346	0.719
Tyzzerella	0.224	0.369	0.605	0.545	0.982	-0.500	0.947
Lachnospiraceae_UCG-	-0.532	0.327	-1.627	0.104	0.753	-1.173	0.109

001							
Lachnospiraceae_UCG-004	0.111	0.509	0.219	0.827	0.991	-0.887	1.109
Colidextribacter	-0.120	0.399	-0.299	0.765	0.991	-0.902	0.663
Flavonifractor	-0.051	0.276	-0.184	0.854	0.991	-0.592	0.490
Oscillibacter	0.038	0.367	0.105	0.917	0.991	-0.681	0.758
Oscillospira	-0.294	0.249	-1.183	0.237	0.932	-0.782	0.193
Anaerotruncus	-0.190	0.223	-0.851	0.395	0.973	-0.626	0.247
Butyricicoccus	-0.474	0.377	-1.259	0.208	0.874	-1.213	0.264
Candidatus Soleaferrea	0.091	0.149	0.607	0.544	0.982	-0.202	0.383
Faecalibacterium	0.099	0.392	0.252	0.801	0.991	-0.670	0.868
Negativibacillus	-0.366	0.190	-1.926	0.054	0.753	-0.738	0.006
Ruminococcus	0.274	0.644	0.424	0.671	0.991	-0.990	1.537
Subdoligranulum	0.010	0.534	0.019	0.985	0.991	-1.036	1.057
UBA1819	-0.100	0.320	-0.312	0.755	0.991	-0.727	0.528
Monoglobus	-0.688	0.485	-1.419	0.156	0.801	-1.639	0.262
Dialister	-0.645	0.465	-1.388	0.165	0.801	-1.556	0.266
Veillonella	0.549	0.633	0.866	0.386	0.973	-0.693	1.790
Bilophila	-0.312	0.420	-0.742	0.458	0.973	-1.136	0.512
Parasutterella	-0.582	0.539	-1.079	0.280	0.948	-1.639	0.475
Sutterella	1.252	0.624	2.005	**0.045***	0.753	0.028	2.475
Escherichia-Shigella	-0.347	0.513	-0.676	0.499	0.982	-1.352	0.658
Haemophilus	0.619	0.618	1.002	0.316	0.948	-0.591	1.830
Akkermansia	-0.082	0.284	-0.289	0.773	0.991	-0.638	0.474

Note. Participants exceeding the cutoff values for either *Inhibitory Self-Control* or *Flexibility* on the BRIEF-P were excluded from the EM-control group. Antibiotic treatment within the last 3 months and sex were used as covariates.

** $p < 0.01$; * $p < 0.05$; beta, coefficient obtained from the ANCOM-BC log-linear model; se, standard error of beta; W, test statistic (beta/se)

Table S24. *Comparison of dietary habits between the ISC-risk (N = 26) and ISC non-risk (N = 231) groups (Brunner–Munzel test, Mann–Whitney U test, Fisher's exact test)*

Items	ISC-risk group	ISC non-risk group	Statistics	*p-value*	*q-value*	Effect size
**Food intake frequency** [a]						
rice or bread	4.73 (0.83)	4.71 (0.77)	0.53 [c]	0.599	0.597	0.52 [f]
unrefined grains	1.27 (0.96)	1.37 (0.92)	-1.52 [c]	0.137	0.295	0.45 [f]
root vegetables	3.35 (1.16)	3.61 (1.07)	-1.05 [c]	0.304	0.434	0.44 [f]
green and yellow vegetables	2.54 (1.07)	3.28 (1.16)	-3.36 [c]	**0.002****	**0.026***	0.32 [f]
light colored vegetables	2.85 (1.16)	3.26 (1.14)	-1.75 [c]	0.090	0.275	0.40 [f]
fruit	3.42 (1.17)	3.40 (1.07)	0.07 [c]	0.943	0.763	0.50 [f]
potatoes	2.46 (0.95)	2.79 (0.89)	-1.64 [c]	0.112	0.284	0.40 [f]
meat	3.58 (0.76)	3.56 (1.01)	0.11 [c]	0.911	0.761	0.51 [f]
seafood	2.54 (0.99)	2.73 (0.92)	-0.97 [c]	0.340	0.434	0.44 [f]
eggs	2.80 (1.22)	3.01 (0.97)	-0.62 [c]	0.541	0.578	0.46 [f]
milk or cheese	3.19 (1.41)	3.66 (1.14)	-1.41 [c]	0.169	0.337	0.41 [f]
yogurt or probiotic drinks	2.88 (1.14)	3.00 (1.05)	-0.59 [c]	0.558	0.578	0.46 [f]
soy products	2.73 (0.96)	2.76 (1.02)	-0.16 [c]	0.874	0.754	0.49 [f]
beans	1.92 (0.98)	2.10 (0.83)	-1.22 [c]	0.232	0.384	0.43 [f]
natto	2.08 (1.06)	1.97 (0.86)	0.24 [c]	0.813	0.726	0.51 [f]
pickled vegetables	1.35 (0.75)	1.34 (0.65)	-0.24 [c]	0.812	0.726	0.49 [f]
seaweed	2.04 (0.92)	2.28 (0.81)	-1.60 [c]	0.121	0.284	0.41 [f]
mushroom	2.38 (0.90)	2.58 (0.95)	-0.87 [c]	0.391	0.460	0.45 [f]
snack	3.69 (1.05)	3.48 (1.05)	0.95 [c]	0.352	0.434	0.56 [f]
instant noodles	1.56 (0.51)	1.35 (0.55)	2.19 [c]	**0.036***	0.274	0.61 [f]
fast food	1.58 (0.50)	1.40 (0.51)	1.77 [c]	0.086	0.275	0.59 [f]
ion supply drink	1.27 (0.60)	1.14 (0.44)	1.01 [c]	0.322	0.434	0.54 [f]
vegetable juice	2.35 (1.16)	2.11 (0.99)	0.97 [c]	0.341	0.434	0.56 [f]
soft drinks	2.12 (1.37)	1.68 (0.87)	1.21 [c]	0.237	0.384	0.57 [f]
**JDI score** [a]	7.19 (2.12)	8.07 (1.69)	1.97 [d]	**0.048***	0.274	0.12 [g]
**Picky eating** [b]	Yes: 17 (65%)	Yes: 75 (33%)	3.88 [e]	**0.002****	**0.026***	0.21 [h]

	No: 9 (35%)<br>Not answered: 0	No: 155 (67%)<br>Not answered: 1				

[a]Mean (SD). [b]N (%). [c] B_N^{BF}. [d]Z. [e]Odds ratio. [f]estimate $\hat{p}^n$. [g]r. [h]Cramer's V
**p or $q < 0.01$; *p or $q < 0.05$

Table S25. *Comparison of physical symptoms between the ISC-risk (N = 26) and the ISC non-risk (N = 231) groups (Mann–Whitney U test, Fisher's exact test)*

Items	ISC-risk group	ISC non-risk group	Statistics	*p*-value	*q*-value	Effect size
Diarrhea frequency [a]	None: 19<br>Once a week: 2<br>Once every 4-5 days: 0<br>Once every 2-3 days: 1<br>Every day: 0<br>Not answered: 4	None: 211<br>Once a week: 2<br>Once every 4-5 days: 2<br>Once every 2-3 days: 4<br>Every day: 5<br>Not answered: 7	1.33 [c]	0.18	0.34	0.54 [e]
Abnormal stool form [b]	Yes: 8 (32%)<br>No: 17 (68%)<br>Not answered: 1	Yes: 37 (17%)<br>No: 185 (83%)<br>Not answered: 9	0.43 [d]	0.10	0.27	0.12 [f]
Urticaria/Atopy [b]	Yes: 3 (12%)<br>No: 23 (88%)<br>Not answered: 0	Yes: 18 (8%)<br>No: 210 (92%)<br>Not answered: 3	1.52 [d]	0.46	0.52	0.04 [f]
Asthma [b]	Yes: 6 (23%)<br>No: 20 (77%)<br>Not answered: 0	Yes: 23 (11%)<br>No: 205 (89%)<br>Not answered: 3	2.66 [d]	0.09	0.27	0.12 [f]
Allergic rhinitis [b]	Yes: 1 (4%)<br>No: 25 (96%)<br>Not answered: 0	Yes: 10 (4%)<br>No: 218 (96%)<br>Not answered: 3	0.87 [d]	1.00	0.78	0.01 [f]
Hay fever [b]	Yes: 3 (12%)<br>No: 23 (88%)<br>Not answered: 0	Yes: 6 (3%)<br>No: 222 (97%)<br>Not answered: 3	4.78 [d]	0.05	0.27	0.15 [f]
Food allergy [b]	Yes: 3 (12%)<br>No: 23 (88%)<br>Not answered: 0	Yes: 21 (9%)<br>No: 210 (91%)<br>Not answered: 0	1.30 [d]	0.72	0.69	0.03 [f]

[a]N. [b]N (%). [c]Z. [d]Odds ratio. [e]r. [f]Cramer's V
** $p < 0.01$; $^{+}q < 0.10$

Table S26. *Comparison of dietary habits between the FL-risk (N = 18) and FL non-risk (N = 239) groups (Brunner–Munzel test, Mann–Whitney U test, Fisher's exact test)*

Items	FL-risk group	FL non-risk group	Statistics	*p*-value	*q*-value	Effect size
**Food intake frequency** [a]						
rice or bread	4.50 (1.15)	4.72 (0.74)	-0.31 [c]	0.759	0.895	0.48 [f]
unrefined grains	1.00 (0.00)	1.39 (0.95)	-7.35 [c]	**3.E-12*** **	**1.E-10*** **	0.41 [f]
root vegetables	3.28 (1.36)	3.61 (1.06)	-0.90 [c]	0.382	0.673	0.43 [f]
green and yellow vegetables	2.89 (1.32)	3.23 (1.16)	-1.10 [c]	0.287	0.591	0.42 [f]
light colored vegetables	3.22 (1.31)	3.21 (1.13)	-0.02 [c]	0.982	1.000	0.50 [f]
fruit	2.94 (1.16)	3.43 (1.06)	-1.78 [c]	0.091	0.377	0.37 [f]
potatoes	2.28 (0.57)	2.79 (0.91)	-3.16 [c]	**0.004** **	**0.072** †	0.34 [f]
meat	3.39 (0.98)	3.58 (0.99)	-0.72 [c]	0.482	0.731	0.46 [f]
seafood	2.39 (0.92)	2.74 (0.92)	-1.62 [c]	0.122	0.419	0.39 [f]
eggs	3.28 (1.13)	2.97 (0.98)	1.38 [c]	0.185	0.469	0.60 [f]
milk or cheese	3.56 (1.34)	3.62 (1.16)	-0.04 [c]	0.965	1.000	0.50 [f]
yogurt or probiotic drinks	2.75 (1.07)	3.01 (1.06)	-0.39 [c]	0.703	0.860	0.47 [f]
soy products	2.44 (0.62)	2.78 (1.03)	-1.80 [c]	0.083	0.377	0.41 [f]
beans	2.00 (0.77)	2.09 (0.85)	-0.26 [c]	0.799	0.909	0.48 [f]
natto	1.89 (0.96)	1.99 (0.88)	-0.64 [c]	0.532	0.731	0.46 [f]
pickled vegetables	1.24 (0.56)	1.35 (0.67)	-0.82 [c]	0.424	0.699	0.46 [f]
seaweed	2.00 (0.84)	2.27 (0.82)	-1.32 [c]	0.204	0.479	0.41 [f]
mushroom	2.39 (1.04)	2.57 (0.94)	-0.88 [c]	0.387	0.673	0.44 [f]
snack	3.67 (1.08)	3.49 (1.05)	0.69 [c]	0.496	0.731	0.55 [f]
instant noodles	1.47 (0.62)	1.36 (0.54)	0.66 [c]	0.518	0.731	0.54 [f]
fast food	1.72 (0.46)	1.39 (0.51)	2.95 [c]	**0.008** **	**0.086** †	0.67 [f]
ion supply drink	1.28 (0.67)	1.14 (0.44)	0.60 [c]	0.557	0.735	0.53 [f]
vegetable juice	2.44 (1.29)	2.11 (0.99)	0.90 [c]	0.378	0.673	0.57 [f]
soft drink	1.94 (0.94)	1.71 (0.94)	1.39 [c]	0.180	0.469	0.59 [f]
**JDI score** [a]	6.94 (1.83)	8.06 (1.73)	2.51 [d]	**0.012***	**0.099**†	0.16 [g]
**Picky eating** [b]	Yes: 6 (33%)	Yes: 86 (36%)	0.88 [e]	1.000	1.000	0.02 [h]

	No: 12 (67%)<br>Not answered: 0	No: 152 (64%)<br>Not answered: 1				

[a]Mean (SD). [b]N (%). [c] B_N^{BF}. [d]Z. [e]Odds ratio. [f]estimate $\hat{p}^n$. [g]r. [h]Cramer's V
***p or $q < 0.001$; **$p < 0.01$; *$p < 0.05$; †$q < 0.10$

Table S27. *Comparison of physical symptoms between the FL-risk (N = 18) and FL non-risk (N = 239) groups (Brunner–Munzel test, Fisher's exact test)*

**Items**	**FL-risk group**	**FL non-risk Group**	**Statistics**	***p*-value**	***q*-value**	**Effect size**
Diarrhea frequency [a]	None: 13<br>Once a week: 1<br>Once every 4-5 days: 0<br>Once every 2-3 days: 1<br>Every day: 1<br>Not answered: 2	None: 217<br>Once a week: 3<br>Once every 4-5 days: 2<br>Once every 2-3 days: 4<br>Every day: 4<br>Not answered: 9	1.28 [c]	0.22	0.48	0.565 [e]
Abnormal stool form [b]	Yes: 3 (18%)<br>No: 14 (82%)<br>Not answered: 1	Yes: 42 (18%)<br>No: 188 (82%)<br>Not answered: 9	1.04 [d]	1.00	1.00	0.004 [f]
Urticaria/Atopy [b]	Yes: 3 (17%)<br>No: 15 (83%)<br>Not answered: 0	Yes: 18 (8%)<br>No: 218 (92%)<br>Not answered: 3	2.41 [d]	0.18	0.47	0.084 [f]
Asthma [b]	Yes: 5 (28%)<br>No: 13 (72%)<br>Not answered: 0	Yes: 24 (10%)<br>No: 212 (90%)<br>Not answered: 3	3.37 [d]	**0.04***	0.22	0.142 [f]
Allergic rhinitis [b]	Yes: 3 (17%)<br>No: 15 (83%)<br>Not answered: 0	Yes: 8 (3%)<br>No: 228 (97%)<br>Not answered: 3	5.63 [d]	**0.03***	0.22	0.167 [f]
Hay fever [b]	Yes: 2 (11%)<br>No: 16 (89%)<br>Not answered: 0	Yes: 7 (3%)<br>No: 229 (97%)<br>Not answered: 3	4.05 [d]	0.13	0.42	0.113 [f]
Food allergy [b]	Yes: 2 (11%)<br>No: 16 (89%)<br>Not answered: 0	Yes: 22 (9%)<br>No: 217 (91%)<br>Not answered: 0	1.23 [d]	0.68	0.86	0.017 [f]

[a]N. [b]N (%). [c] B_N^{BF}. [d]Odds ratio. [e]estimate $\hat{p}^n$. [f]Cramer's V
*$p < 0.05$; †$q < 0.10$

Table S28. *Comparison of dietary habits between the EM-risk (N = 20) and EM non-risk (N = 237) groups (Brunner–Munzel test, Mann–Whitney U test, Fisher's exact test)*

Items	EM-risk group	EM non-risk group	Statistics	*p*-value	*q*-value	Effect size
**Food intake frequency** [a]						
rice or bread	4.70 (0.92)	4.71 (0.76)	0.60 [c]	0.56	0.82	0.52 [f]
unrefined grains	1.25 (0.91)	1.37 (0.92)	-1.03 [c]	0.31	0.82	0.46 [f]
root vegetables	3.65 (1.14)	3.58 (1.08)	0.41 [c]	0.68	0.82	0.53 [f]
green and yellow vegetables	3.10 (1.12)	3.21 (1.17)	-0.42 [c]	0.68	0.82	0.47 [f]
light colored vegetables	2.95 (1.00)	3.24 (1.15)	-1.28 [c]	0.21	0.82	0.42 [f]
fruit	3.35 (1.27)	3.40 (1.06)	-0.18 [c]	0.86	0.87	0.49 [f]
potatoes	2.80 (0.95)	2.75 (0.90)	0.26 [c]	0.80	0.87	0.52 [f]
meat	3.40 (1.05)	3.58 (0.99)	-0.66 [c]	0.52	0.82	0.46 [f]
seafood	2.80 (0.77)	2.71 (0.94)	0.52 [c]	0.61	0.82	0.53 [f]
eggs	3.00 (1.08)	2.99 (0.99)	0.11 [c]	0.91	0.87	0.51 [f]
milk or cheese	3.65 (1.23)	3.61 (1.17)	0.09 [c]	0.93	0.87	0.51 [f]
yogurt or probiotic drinks	2.75 (1.07)	3.01 (1.06)	-1.15 [c]	0.26	0.82	0.42 [f]
soy products	2.55 (0.60)	2.77 (1.04)	-1.07 [c]	0.29	0.82	0.45 [f]
beans	2.05 (0.51)	2.09 (0.87)	0.10 [c]	0.92	0.87	0.50 [f]
natto	1.80 (0.62)	2.00 (0.90)	-0.77 [c]	0.45	0.82	0.46 [f]
pickled vegetables	1.20 (0.41)	1.35 (0.68)	-0.83 [c]	0.41	0.82	0.46 [f]
seaweed	1.95 (0.60)	2.28 (0.83)	-1.93 [c]	0.07	0.82	0.40 [f]
mushroom	2.40 (0.68)	2.57 (0.96)	-0.83 [c]	0.41	0.82	0.46 [f]
snack	3.48 (1.05)	3.75 (0.97)	1.10 [c]	0.28	0.82	0.57 [f]
instant noodles	1.47 (0.61)	1.36 (0.54)	0.77 [c]	0.45	0.82	0.55 [f]
fast food	1.55 (0.51)	1.4 (0.51)	1.27 [c]	0.22	0.82	0.57 [f]
ion supply drink	1.35 (0.81)	1.14 (0.41)	0.96 [c]	0.35	0.82	0.55 [f]
vegetable juice	2.20 (1.11)	2.13 (1.01)	0.22 [c]	0.83	0.87	0.51 [f]
soft drink	1.80 (0.95)	1.72 (0.94)	0.46 [c]	0.65	0.82	0.53 [f]
**JDI score** [a]	8.15 (1.69)	7.97 (1.76)	0.25 [d]	0.80	0.87	0.02 [g]
**Picky eating** [b]	Yes: 10 (50%)<br>No: 10 (50%)	Yes: 82 (35%)<br>No: 154 (65%)	1.87 [e]	0.22	0.82	0.09 [h]

	Not answered: 0	Not answered: 1				

[a]Mean (SD). [b]N (%). [c] B_N^{BF}. [d]Z. [e]Odds ratio. [f]estimate $\hat{p}^n$. [g]r. [h]Cramer's V

Table S29. *Comparison of physical symptoms between the EM-risk (N = 20) and EM non-risk (N = 237) groups (Brunner–Munzel test, Fisher's exact test)*

**Items**	**EM-risk group**	**EM non-risk group**	**Statistics**	***p*-value**	***q*-value**	**Effect size**
Diarrhea frequency [a]	None: 15<br>Once a week: 1<br>Once every 4-5 days: 1<br>Once every 2-3 days: 1<br>Every day: 1<br>Not answered: 1	None: 211<br>Once a week: 3<br>Once every 4-5 days: 1<br>Once every 2-3 days: 4<br>Every day: 4<br>Not answered: 3	1.62 [c]	0.12	0.82	0.58 [e]
Abnormal stool form [b]	Yes: 5 (25%)<br>No: 15 (75%)<br>Not answered: 0	Yes: 40 (18%)<br>No: 187 (82%)<br>Not answered: 10	0.64 [d]	0.38	0.82	0.05 [f]
Urticaria/Atopy [b]	Yes: 2 (10%)<br>No: 18 (90%)<br>Not answered: 0	Yes: 19 (8%)<br>No: 215 (92%)<br>Not answered: 3	1.26 [d]	0.67	0.82	0.02 [f]
Asthma [b]	Yes: 4 (20%)<br>No: 16 (80%)<br>Not answered: 0	Yes: 25 (11%)<br>No: 209 (89%)<br>Not answered: 3	2.08 [d]	0.26	0.82	0.08 [f]
Allergic rhinitis [b]	Yes: 1 (4%)<br>No: 19 (96%)<br>Not answered: 0	Yes: 10 (4%)<br>No: 224 (96%)<br>Not answered: 3	1.18 [d]	0.60	0.82	0.01 [f]
Hay fever [b]	Yes: 1 (5%)<br>No: 19 (95%)<br>Not answered: 0	Yes: 8 (3%)<br>No: 226 (97%)<br>Not answered: 3	1.48 [d]	0.53	0.82	0.02 [f]
Food allergy [b]	Yes: 2 (10%)<br>No: 18 (90%)<br>Not answered: 0	Yes: 22 (9%)<br>No: 215 (91%)<br>Not answered: 0	1.09 [d]	1.00	0.91	0.01 [f]

[a]N. [b]N (%). [c] B_N^{BF}. [d]Odds ratio. [e]estimate $\hat{p}^n$. [f]Cramer's V
